# Radon concentration in seawater as a geochemical indicator of submarine fault activity in the Yatsushiro Sea, Japan

**DOI:** 10.1038/s41598-024-59006-6

**Published:** 2024-04-15

**Authors:** Kuniyo Kawabata, Fumiaki Tsunomori, Yujin Kitamura, Yen-Yu Lin, Chung-Han Chan, Kuo-Fong Ma

**Affiliations:** 1 Center for General Education, Kagoshima University, 1-21-30 Korimoto, Kagoshima-City, Kagoshima 890-0082 Japan; 2 Geochemical Research Center, Graduate School of Science, The University of Tokyo, 7-3-1 Hongo, Bunkyo-ku, Tokyo 113-0033 Japan; 3 Graduate School of Science and Engineering, Kagoshima University, 1-21-35 Korimoto, Kagoshima-City, Kagoshima 890-0082 Japan; 4 Department of Earth Sciences, National Central University, No.300, Zhongda Rd., Zhongli, Taoyuan 320317 Taiwan; 5 Earthquake-Disaster and Risk Evaluation and Management (E-DREaM) Center, National Central University, No.300, Zhongda Rd., Zhongli, Taoyuan 320317 Taiwan; 6 Graduate Institute of Applied Geology, National Central University, No.300, Zhongda Rd., Zhongli, Taoyuan 320317 Taiwan; 7 Institute of Earth Sciences, Academia Sinica, 128, Sec. 2, Academia Road, Nangang, Taipei 11529 Taiwan

**Keywords:** Hydrology, Natural hazards, Ocean sciences, Solid Earth sciences

## Abstract

This study examined the relationship between radon (^222^Rn) concentrations in seawater and crustal activity in the Yatsushiro Sea by investigating the submarine fault zone situated at the southern end of the Futagawa–Hinagu fault zone, activated by the 2016 Kumamoto earthquake (M7.3). We conducted an analysis of ^222^Rn concentration in samples of bottom water just above the seafloor and pore water in sediments, utilizing multiple and piston cores from the *Hakuho Maru* Expedition KH18-3. The findings revealed significantly elevated ^222^Rn concentrations in the central sites of the Yatsushiro Sea, coinciding with a high-stress field exhibiting dense active faults. Seismicity analysis revealed heightened moment release and a low b-value post the 2016 Kumamoto earthquake, indicative of increased seismic activity and the potential for substantial earthquakes in the Yatsushiro Sea vicinity. Our results indicate that heightened concentrations of ^222^Rn in seawater can serve as an effective tracer for identifying and estimating submarine fault activities. Moreover, our research highlights the utility of ^222^Rn concentrations in detecting active submarine faults and assessing their activity. It contributes to a comprehensive understanding of the potential for significant earthquakes in the Yatsushiro Sea in the future.

## Introduction

Radon-222 (^222^Rn) is a naturally occurring radioactive noble gas, formed in an intermediate step in the radioactive uranium decay chain. ^222^Rn is highly soluble in water, which allows it to move freely with groundwater and fluids within the Earth's crust. The release rate of ^222^Rn is primarily contingent on radium-226 concentration in rocks and the surface area of rocks/minerals^1^. Variations in surface area, influenced by factors such as porosity, cracks, and fractures, impact the contact area between pore fluid (water or air) and rock, thereby influencing the release of ^222^Rn. Consequently, the ^222^Rn concentration can serve as a tracer for determining subsurface rock types and providing insights into groundwater aquifers.

^222^Rn has been recognized as a precursor of earthquakes and an indicator of crustal deformation^2–7^. Recently, monitoring networks have been established for soil ^222^Rn in Italy and China. Machine learning techniques have been applied to analyze the collected data, marking a recent advancement^8,9^. Active faults, often maturing into well-defined structures with gouge in the fault core, cataclasite, and fracture zones, exhibit an exceptionally high specific surface area in the damaged zone, e.g., 3.3 × 10^6^ m^2^ per unit area of the fault surface^10^. Water in the crust, such as groundwater and hot springs selectively traverse permeable zones, turning fault zones into conduits for groundwater. The concentration of ^222^Rn in the crust, especially in groundwater, is a powerful indicator of active faults, allowing for the estimation of fault activity. By mapping ^222^Rn in groundwater, Tsunomori^11^ identified large active faults and concluded that the faults may be larger than previously estimated. Furthermore, a correlation between ^222^Rn concentration and stress evolution was discerned through spatiotemporal variations before and after a large earthquake^12^. These investigations mapped groundwater ^222^Rn concentration on land, revealing active fault locations and estimating their activity. However, the variations in ^222^Rn concentration around submarine faults have never been reported or discussed. In this study, we attempted to generate a ^222^Rn concentration map for the Yatsushiro submarine faults, believed to have been activated by the 2016 Kumamoto earthquake. This report presents the ^222^Rn concentration findings and discusses the relationship between ^222^Rn concentration and seismicity in the vicinity of the submarine faults.

## Yatsushiro submarine faults and sampling

**Figure 1:**
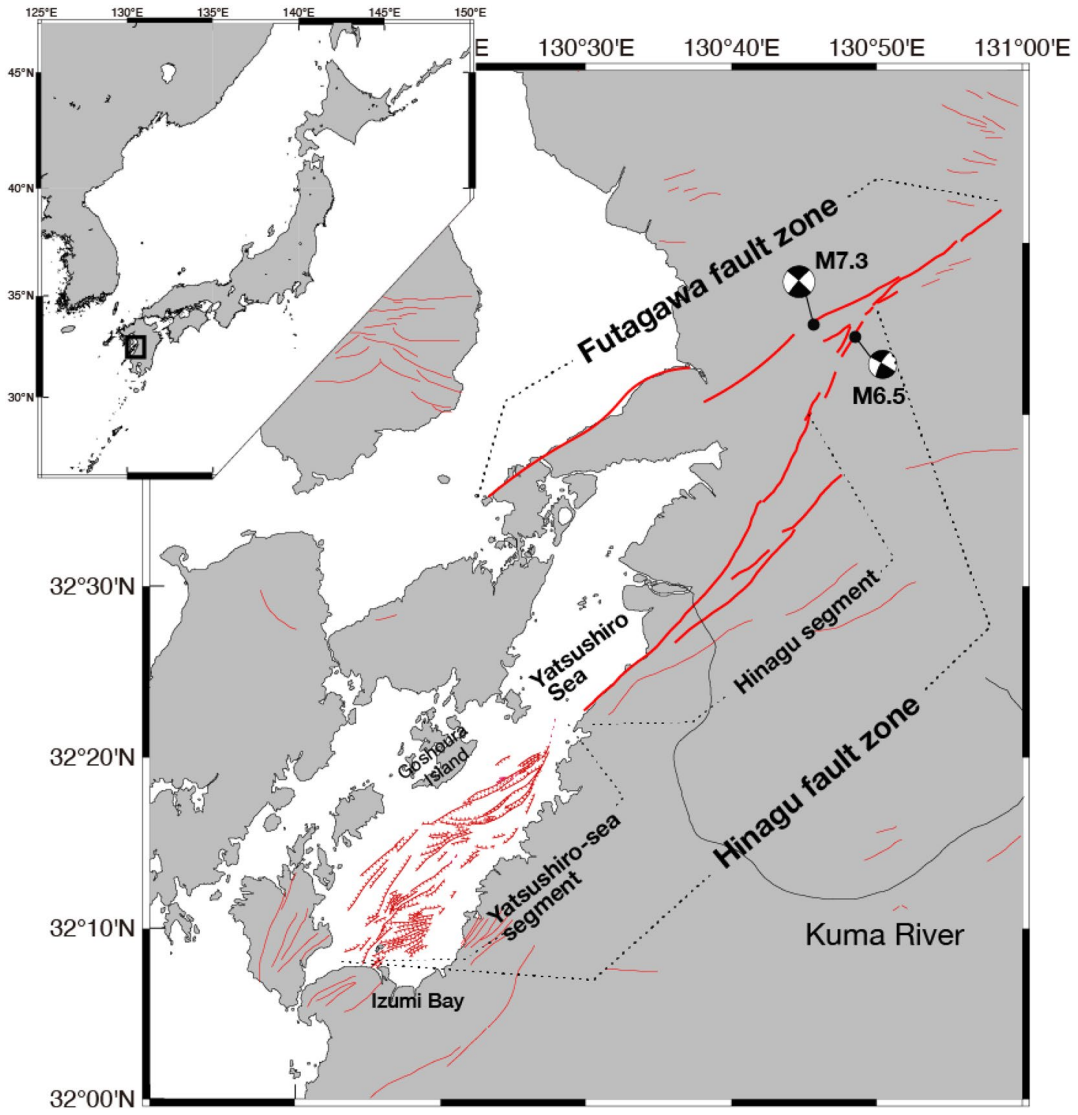
Map of active faults around the Yatsushiro Sea. Red solid and dashed lines show active faults on land and submarine active faults, respectively. Black circles with focal mechanism solutions represent the epicenters of the 2016 Kumamoto earthquakes. The map was generated using Generic Mapping Tools (GMT), ver.6.5^28^.

The Yatsushiro submarine faults constitute an active fault group situated in the Yatsushiro Sea, southwest of Kyushu, Japan. This fault network represents the southernmost segment of the Futagawa-Hinagu fault zone, the epicenter of the 2016 Kumamoto Earthquake^13,14^ (Fig. 1). Earthquakes larger than M6.0 have repeatedly occurred in this Yatsushiro Sea segment, raising concerns regarding the potential hazard of another large seismic event in the near future^15^.

Historical seismic activities in the Yatsushiro Sea segment date back to 744 and 1619 AD^16,17^, documented through seafloor acoustic surveys and sedimentary rock dating^14,18^. The Yatsushiro submarine faults exhibit a steep to nearly vertical orientation, with discernible folding structures in the jog sections connecting the Yatsushiro Sea segment to the adjacent Hinagu segment^18^. A seismicity pattern analysis post the 2016 Kumamoto earthquake indicates that the jog section is under high-stress status^19^, suggesting an elevated potential for a large earthquake in the area, given the high seismicity rate and active crustal deformation.

The Hakuho Maru Expedition KH18-3 research cruise, conducted from July 27th to 30th, 2018, aimed to elucidate seafloor environmental changes resulting from active faults and submarine landslides triggered by the 2016 Kumamoto Earthquake in the Yatsushiro Sea. Additionally, the expedition sought to create a ^222^Rn concentration map for the Yatsushiro submarine faults. During the expedition, we collected two types of cores using a multiple corer and a piston corer, strategically positioned directly above or near active submarine faults. The multiple corer retrieved sediments below the seafloor in one-meter tubes, accompanied by seawater just above the seafloor, referred to as bottom water. Upon retrieval of the multiple corer onto the deck, the seawater was sampled to measure the ^222^Rn concentration. For deeper sediment sampling, a long piston corer was employed. In the piston cores, we collected upwelling pore water, which was obtained by self-loading and accumulated at the core's top after standing for a day. Subsequently, the ^222^Rn concentrations in the piston core water were measured.

## Seismicity around Yatsushiro Sea

**Figure 2:**
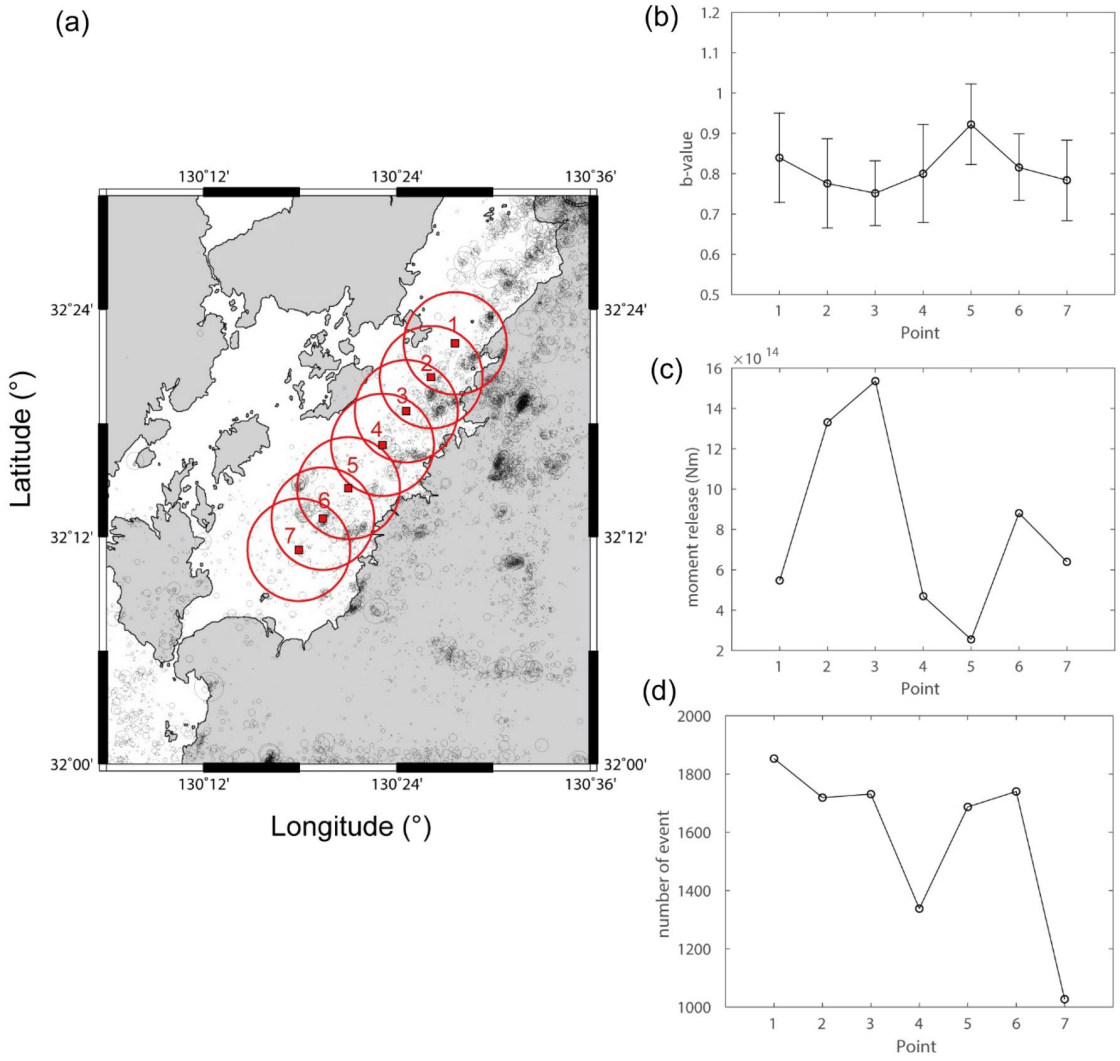
(**a**) Regions where the seismicity study was conducted. The red circles indicate the six regions for the seismicity investigation along the Yatsushiro Sea. Analysis results showcasing (**b**) b-value, (**c**) moment release, and (**d**) event count for each specified region. The map was prepared using GMT, ver.6.5^28^.

To analyze the seismicity in the Yatsushiro Sea, earthquake parameters (event location and magnitude) were collected from the earthquake catalog of the Japan Meteorological Agency spanning from January 1996 to February 2020. Seven circular observation regions (regions 1–7) were delineated along the central axis of the Yatsushiro Sea, each possessing a 5 km radius (Fig. 2a). These circular regions exhibited an approximate 5-km overlap in radius with their nearest counterparts, collectively covering the majority of the Yatsushiro Sea. A total of 1853, 1719, 1731, 1338, 1687, 1740, and 1027 earthquakes were incorporated into our calculations for regions 1–7, respectively.

Utilizing this dataset, the *b*-value and moment release for the specified regions were determined, as outlined in the Method section. Figures 2b, c and d, along with S1, summarize the *b*-value, moment release, and event count over the entire period. Regions 2 and 3 exhibited lower *b*-values and higher moment release. Given the substantial seismic event count in each zone, the associated uncertainty in *b*-value calculation is comparatively low. Specifically, when considering both the *b*-value and event count, the standard deviation of the *b*-value ranges from 0.018 to 0.024, a range significantly smaller than the temporal variations observed in *b*-values. Notably, the central area in the Yatsushiro Sea, encompassing regions 2 and 3, displayed heightened seismic activity. These results suggest that regions 2 and 3 may possess heightened potential for stress accumulation.

**Figure 3:**
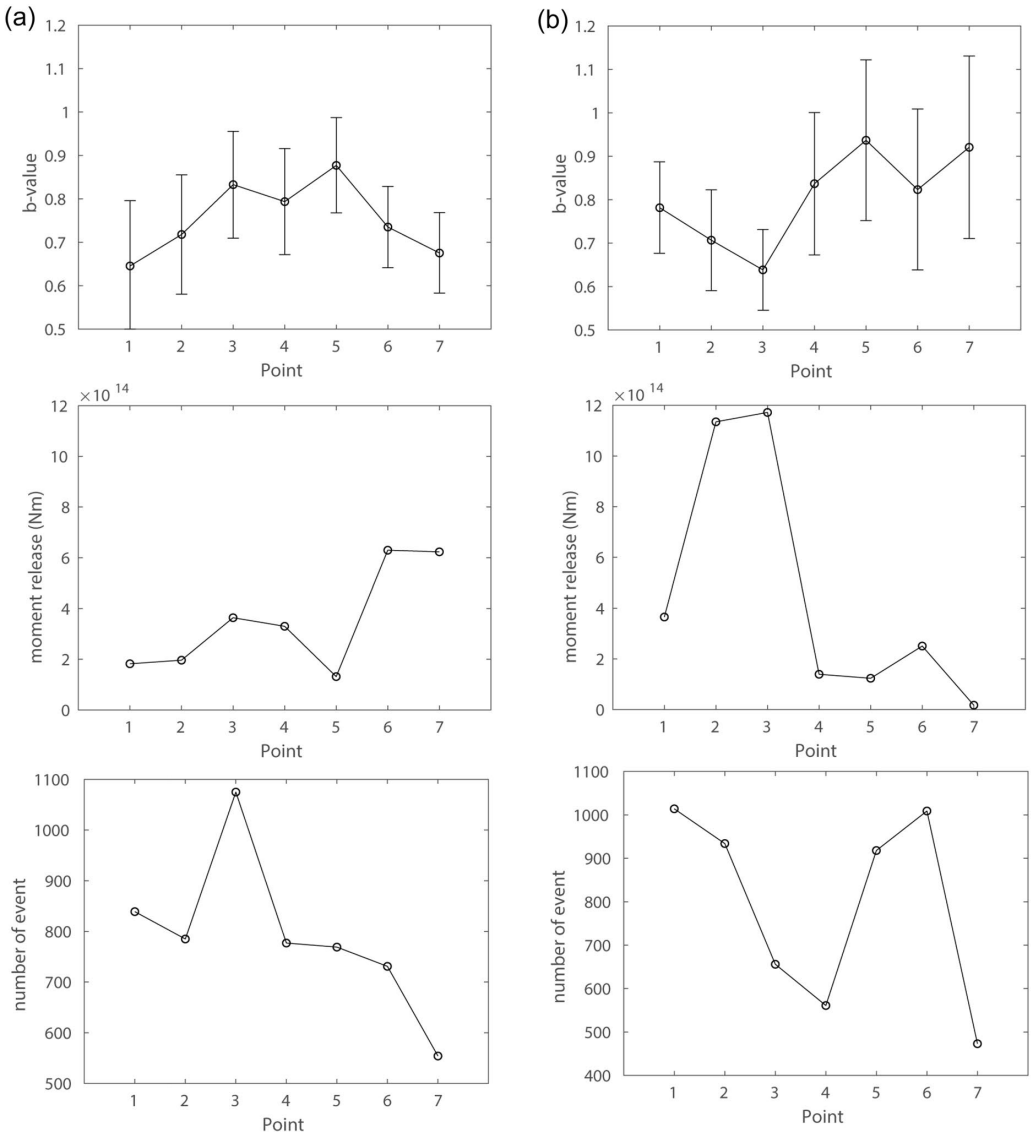
Analysis results showcasing b-value, moment release, and event count for each specified area (**a**) before and (**b**) after the 2016 Kumamoto earthquake.

Further analysis was conducted on seismic data pre and post the 2016 Kumamoto earthquake (Figs. 3, S2, S3). Following the earthquake, there were noticeable changes in the spatial distribution of *b*-value and moment release were observed. Specifically, *b*-values in regions 2 and 3 decreased post-earthquake. A higher moment release was observed in the southern area (regions 6 and 7) before the earthquake, whereas post-earthquake, the central area (regions 2 and 3) exhibited heightened moment release. These findings indicate an intensified crustal deformation process, including increased seismic activity in the central area of the Yatsushiro Sea, subsequent to the 2016 Kumamoto earthquake.

## Results for core analysis and ^222^Rn concentrations

**Figure 4:**
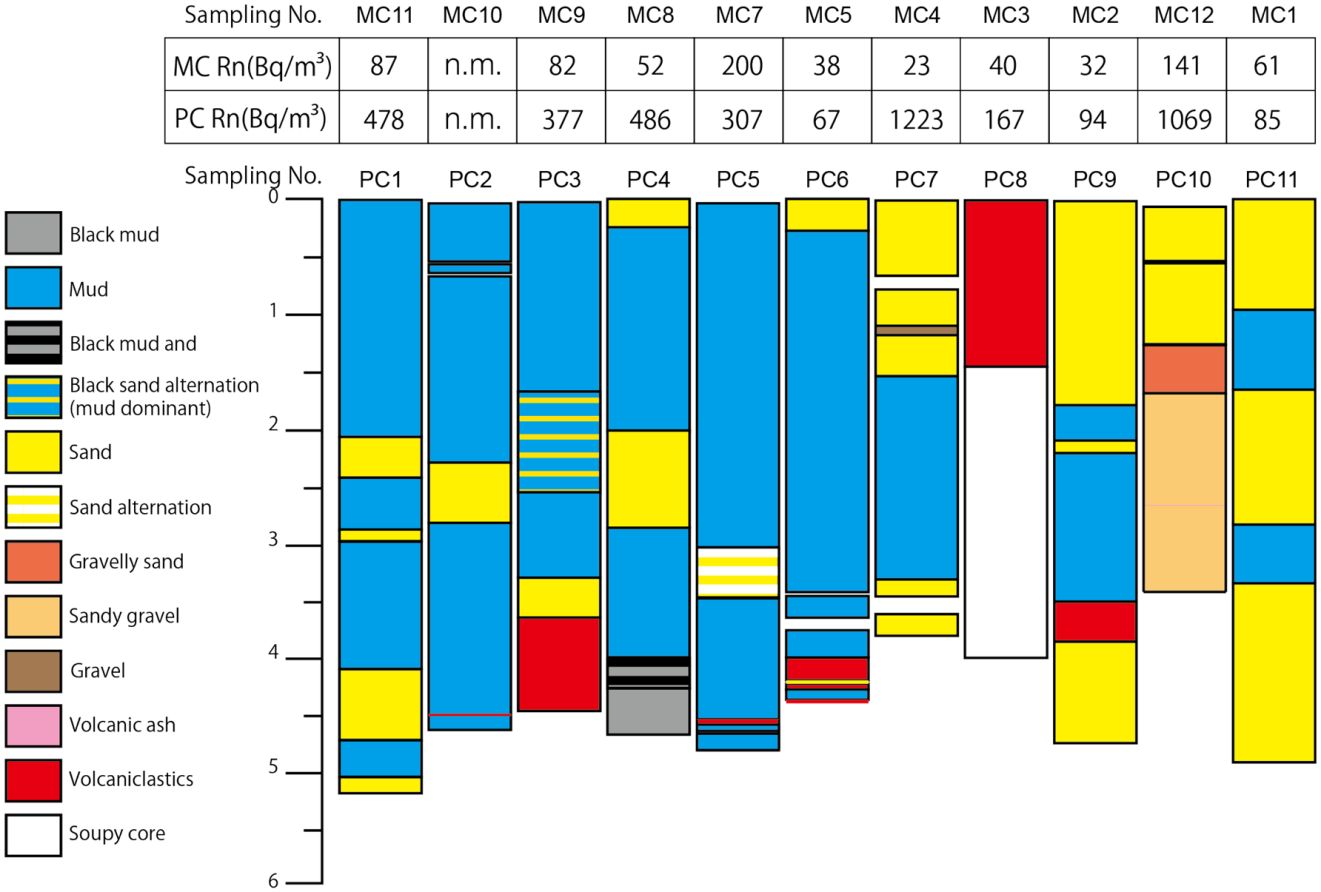
Geological columnar sections of piston core (PC) and corresponding ^222^Rn concentrations (PC samples) and bottom water samples from nearly the same location (multiple core (MC) samples). “n.m.” indicates that the measurement was not conducted due to the inability to obtain the seawater samples. Conglomerate layers are observed in cores from sites PC7 and PC10.

Results obtained from the examination of piston cores are depicted in Fig. 4 and Supplementary Fig S4. The sediment composition in the Yatsushiro Sea primarily includes mud and sand, along with some volcaniclastics. In the southwestern region of the Yatsushiro Sea, conglomerate beds were observed at sites PC7 and PC10 (Fig. S4 a–e). Cores, especially those with conglomerate layers, exhibit a higher presence of upwelling water. Within the conglomerate layer of PC 10, minor granitoid gravels were detected, predominantly consisting of sandy gravels. These granules likely originate from Goshoura Island, located to the northwest of PC10, where Granodiorite is partially exposed in sedimentary rocks. Sediments obtained by multiple cores consist entirely of soft mud (Fig. S4 f.). The mud collected from all sampling locations is similar.

**Figure 5:**
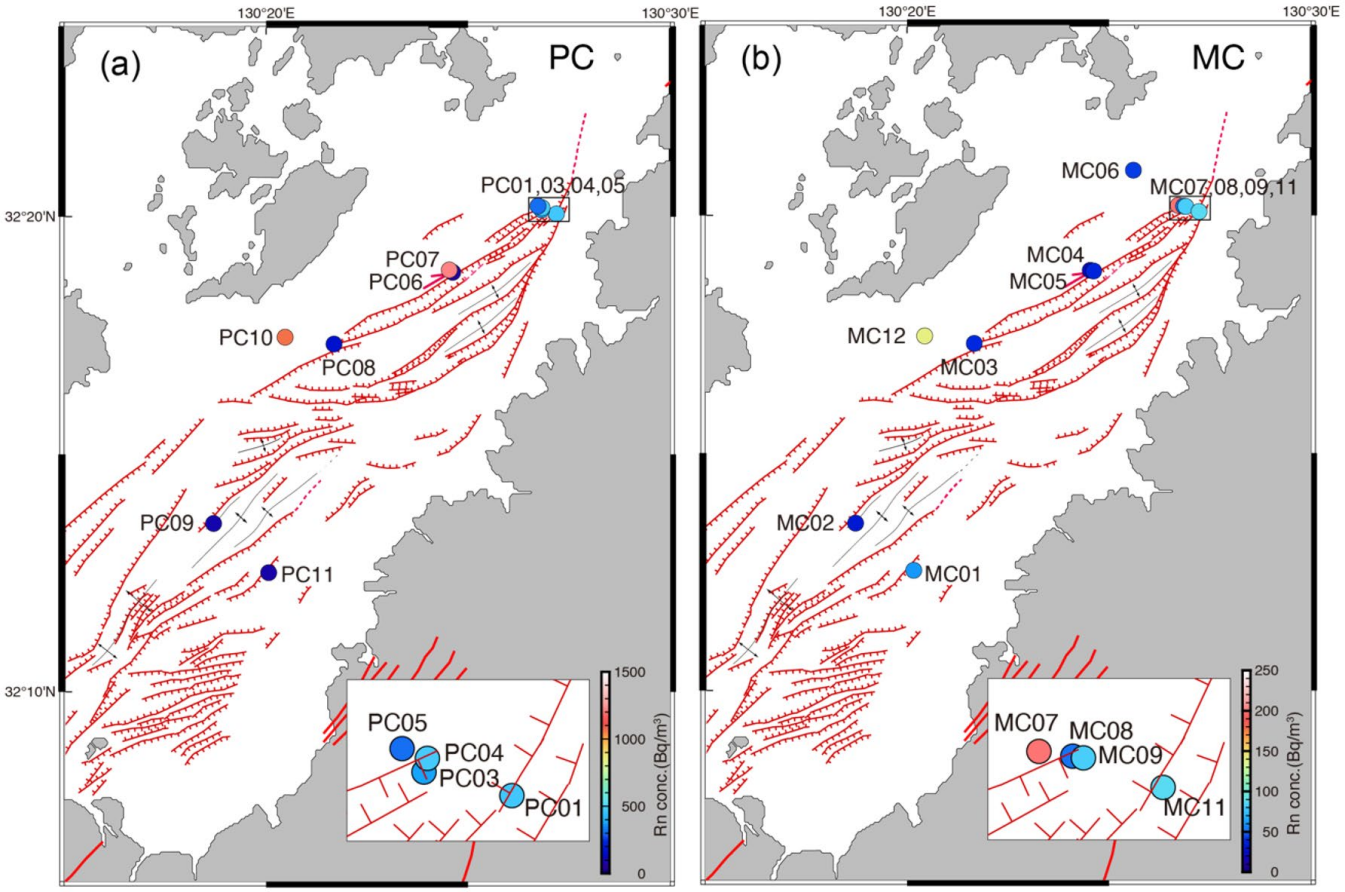
Results of ^222^Rn concentration measurements around Yatsushiro submarine faults^14^ in (**a**) upwelling water from piston core (PC) and (**b**) bottom water from multiple core (MC). The map was generated using GMT, ver.6.5^28^.

The concentration of ^222^Rn in seawater samples obtained using multiple cores (MC) and piston cores (PC) was measured. ^222^Rn concentrations were determined using a radon and thron monitor (RTM 1688, SARAD GmbH, Germany) attached to a gas–water separation bottle (Fig. S5). The detailed measurement technique is discussed in the Methods section.

Results of ^222^Rn concentrations for the MC and PC samples are illustrated in Fig. 5a and b and Table S1. The ^222^Rn concentrations in the PC samples surpassed those in the MC samples, with notably higher concentrations in sediments containing conglomerate layers.

Typically, ^222^Rn is rarely detected in seawater. However, we observed ^222^Rn concentrations in bottom water (MC samples), particularly at sites MC7, 8, 9, 11, and 12, where relatively high ^222^Rn concentrations were noted. MC12 and PC10 represent the same sites, both containing a thick conglomerate layer. The central area in the Yatsushiro Sea around sites MC7, 8, 9, and 11 is a region exhibiting high ^222^Rn concentrations despite the absence of conglomerate beds. The high ^222^Rn concentration regions around MC7, 8, 9, and 11 align with the area seismically most active (2 and 3). The area is situated close to the segment boundary between the Yatsushiro Sea segment and the Hinagu segment (Fig. 1), and the region is considered to be under high stress^18,19^. The high-stress condition can also be inferred from geological structures such as pressure ridge formations and their displacement by active faults in the area^18^.

## Discussion

High concentrations of ^222^Rn in bottom water were observed at sites above conglomerate beds and in high-stress condition areas near the boundary between the Yatsushiro Sea and the Hinagu segments. Various factors may influence the ^222^Rn concentration in bottom water, including (1) different radium concentrations in rock types, (2) water flow from terrestrial sources, (3) water flow rates from sediments, and (4) crustal deformation involving changes in porosity and/or fracture formation^1^.

The amount of radium-226, which decays into ^222^Rn, in host sediments varies based on mineral composition. However, the sediments beneath the Yatsushiro Sea are quasi-homogeneous^20^, with a minor presence of granitic rock only in the gravel layer of PC10, which could contain a relatively high concentration of uranium-238. Therefore, the observation results suggest that the higher ^222^Rn concentration is not solely attributed to differences in rock types. The sample containing a conglomerate layer, PC10, may slightly elevate ^222^Rn concentration.

The primary source of terrestrial water in the region is the Kuma River, the only major river flowing into the Yatsushiro Sea (Fig. 1). Nikpeyman et al.^21^ noted that the ^222^Rn concentration in water discharged from the Kuma River is relatively high along the coastal region near the estuary of the Kuma River in the Yatsushiro Sea. Higher ^222^Rn concentrations were observed in Izumi Bay on the southern side of the Yatsushiro Sea, attributed to submarine groundwater discharge (SGD). However, the offshore regions of the Yatsushiro Sea show minimal impact from ^222^Rn originating from both the Kuma River and SGD in Izumi Bay. In addition to measuring ^222^Rn concentrations, they conducted simulations to assess the seasonal influence of ^222^Rn, including the spread of river water discharging from the sea surface to the seabed. Simulation results confirm that ^222^Rn from the river predominantly affects the northern area of the Yatsushiro Sea. Our sampling locations, situated in the central and southern regions of the Yatsushiro Sea at a depth of around 50 m, receive negligible ^222^Rn contribution from terrestrial water.

Crustal deformation plays a pivotal role in the release of ^222^Rn through alterations in permeability and specific surface area induced by crack formation and fracturing. The fracture zone, encompassing cracks and fractured grains, exhibits a higher water–rock contact area compared to the host rock^22^. Fault zones with flowing water display elevated ^222^Rn concentrations due to increased release from rocks into the surrounding water^11^. Permeability changes resulting from stress-induced crack formation can also influence the flow rate of upwelling water, leading to the discharge of highly concentrated ^222^Rn water onto the seafloor.

Both MC and PC samples indicate that conglomerate beds tend to induce higher ^222^Rn concentrations. These conglomerate layers, characterized by high porosity and increased water content in the pore space, undergo compaction, facilitating the easy upwelling of pore water with elevated concentrations of ^222^Rn. The observed ^222^Rn concentration and related results align with water flowing through porous conglomerate sediments. The flow rate in conglomerates, with higher permeability and porosity compared to muddy and sandy sediments, would be faster. The steady state concentration of ^222^Rn in bottom water at the seafloor is maintained by inflow from sediments, outflow due to migration and diffusion in the bottom water, and its decay. The faster flow of a larger volume of pore water through conglomerates results in a higher supply of ^222^Rn to bottom water than in sediments without conglomerates. The ^222^Rn concentration at site MC 12, including conglomerates, would be higher due to faster upwelling through porous sediments. Minor granitoid gravels around PC10 and MC12 may also contribute to increased ^222^Rn levels.

In the study area, elevated ^222^Rn concentrations were observed in the vicinity of sites MC 7, 8, 9, and 11. Sediments around these sites primarily consist of sand and mud, with no conglomerates. ^222^Rn from river water is negligible around the area. However, the region with higher ^222^Rn concentration aligns with a large number of seismic events, high moment releases and low b-values (Fig. 2), indicative of seismically active and higher stress concentration. Continuous release of ^222^Rn by seismic events, passing through a highly permeable fault zone with water to the seafloor, could contribute to the observed ^222^Rn levels. Following the 2016 Kumamoto earthquake, a notable increase in both the number of seismic events and moment release occurred in the vicinity (Fig. 3). Considering the half-life of ^222^Rn (3.8 days), our measurements reflect the crustal condition after the 2016 Kumamoto earthquake. The heightened seismic activity likely played a role in the increased in-water ^222^Rn levels observed in the central area of the Yatsushiro Sea, specifically around sites MC 7 through 11, suggesting a potential post-earthquake influence on ^222^Rn concentration in the region.

This study establishes the relationship between crustal activity and ^222^Rn concentration. To further validate the correlation between seismic activity and ^222^Rn levels, regular measurements of radon concentration are imperative. Ongoing monitoring will contribute to a comprehensive understanding of the dynamic interplay between crustal processes and ^222^Rn release in marine environments.

## Conclusion

The investigation of ^222^Rn concentrations in the bottom water of the Yatsushiro submarine faults has unveiled elevated levels in highly permeable sediments and regions characterized by intense tectonic and seismic activity. An examination of minor seismic events in the Yatsushiro Sea post the 2016 Kumamoto earthquake indicates a heightened seismic activity. It is evident that seismic events led to the release of ^222^Rn through crack formations, with subsequent movement and upwelling occurring through permeable active faults. Furthermore, the active fault zone itself plays a role in the presence of ^222^Rn in the passing seawater. These discoveries posit that ^222^Rn concentrations can serve as an effective indicator of crustal activity not solely on land but also beneath the sea.

## Methods

### Measurement of in-water ^222^Rn concentration

The methodology employed for determining the ^222^Rn concentration in water aligns with that detailed by Kawabata et al.^12^ Initially, a 1.0-L volume of seawater was transferred into a 2.2-L bottle, which was subsequently sealed with a screwcap housing two tube connectors (see Fig. S1). To expedite the establishment of gas–liquid equilibrium, the bottled seawater underwent vigorous hand agitation for a duration of three minutes. Following this, the air in the gas phase within the bottle was dehumidified utilizing a DRIERITE® drying column and was then directed into a ^222^Rn monitor via an internal pump. The ^222^Rn monitor continuously acquired the energy spectrum of alpha particles emitted by daughter elements of radon-222 over a 15-min period for each measurement. Three consecutive sets of measurements were conducted for each sample, with the maximum values from the second or third measurements deemed as the ^222^Rn concentration data in the gas phase. This approach accounted for incomplete equilibrium conditions during the initial measurement stage.

We estimated the ^222^Rn concentration in the aqueous phase by considering the time elapsed from the date of sampling. The equation for the conservation of ^222^Rn in gas and water in the measurement system is:

1 $${C}_{a}{V}_{a}+{C}_{w}{V}_{w}={C}_{a}\left(eq\right){V}_{a}+{C}_{w}\left(eq\right){V}_{w},$$

where *C*_*a*_ and *C*_*w*_ denote the ^222^Rn concentrations in the gas phase and water phase, respectively, before agitation, and *C*_*a*_(*eq*) and *C*_*w*_(*eq*) are the ^222^Rn concentrations in the gas and water phases at equilibrium, respectively; *V*_*a*_ and *V*_*w*_ represent the volumes of the gas and water phase, respectively.

The ^222^Rn equilibrium constant^23^, *Kd* is expressed as:

2 $$Kd=\frac{{C}_{w}\left(eq\right)}{{C}_{a}\left(eq\right)}=0.15+0.403{\text{exp}}(-0.0502 T)$$

where, by assuming *C*_*a*_ to be 0, we estimated the ^222^Rn concentration in water prior to measurement (*C*_*w*_) using Eq. (1):

3 $${C}_{w}={C}_{a}(eq)\left(\frac{{V}_{a}}{{V}_{w}}+Kd\right)$$

We calculated ^222^Rn concentration in water at sampling time, *C*_*w*0_, using the 3.8 d half-life of ^222^Rn, when measurement start time deviated from the sampling time.

### Analysis of seismicity (*b*-value and moment release)

In our investigation, the determination of the *b*-value in the study regions followed the Gutenberg–Richter relation^24^. The frequency-magnitude distribution can be depicted in a seismogenic domain as:

4 $${log}_{10}N=a-bM,$$

Here, *M* represents the magnitude, *N* indicates the number of earthquakes with a magnitude within *M* ± 0.1, and both *a* and *b* are constants determined through regression analysis of seismic activity. The 90% confidence interval for the slope coefficient was interpreted as the uncertainty of the *b*-value. This parameter signifies the proportion of small to large seismic events, with its variation within a region often reflecting the specific characteristics of its seismic activity. A low *b*-value in a region suggests significant differential stress, indicating proximity to the end of its seismic cycle^25^.

To accurately determine the standard deviation of the b-value, we employed a methodology^26^ that entails the following steps:

5 $$\sigma = bN^{{ - {1}/{2}}} ,$$

where *N* represents the total number of seismic events utilized for estimating the *b*-value. Upon examining the *b*-values depicted in Fig. 2b and the event counts shown in Fig. 2d across regions 1–7, the respective standard deviations (*σ*) are calculated to be 0.020, 0.019, 0.018, 0.022, 0.022, 0.020, and 0.024, respectively.

Furthermore, we calculated seismic moment of all events in each region and added them up to obtain the moment release. The magnitude-moment relation is

6 $${M}_{w}=\left(\frac{l{og}_{10}{M}_{0}}{1.5}\right)-10.73,$$

where *M*_w_ is moment magnitude and *M*_0_ is seismic moment in dyne-cm^27^.

### Supplementary Information

Supplementary Figures.

Supplementary Table S1.

## Data Availability

All data generated or analyzed during this study are included in this published article (and its Supplementary Information file). The earthquake parameters were collected from the earthquake catalog of the Japan Meteorological Agency (https://www.data.jma.go.jp/svd/eqev/data/bulletin/hypo_e.html).
