# Supplementary material for: Radon concentration in seawater as a geochemical indicator of submarine fault activity in the Yatsushiro Sea, Japan
Source: Sci Rep. 2024 Apr 15;14:8664. doi: 10.1038/s41598-024-59006-6 (PMC11018784; doi:10.1038/s41598-024-59006-6)
Supplement: Supplementary file 1 — Supplementary Figures. [file 41598_2024_59006_MOESM1_ESM.docx]

**Supplementary figures and table**


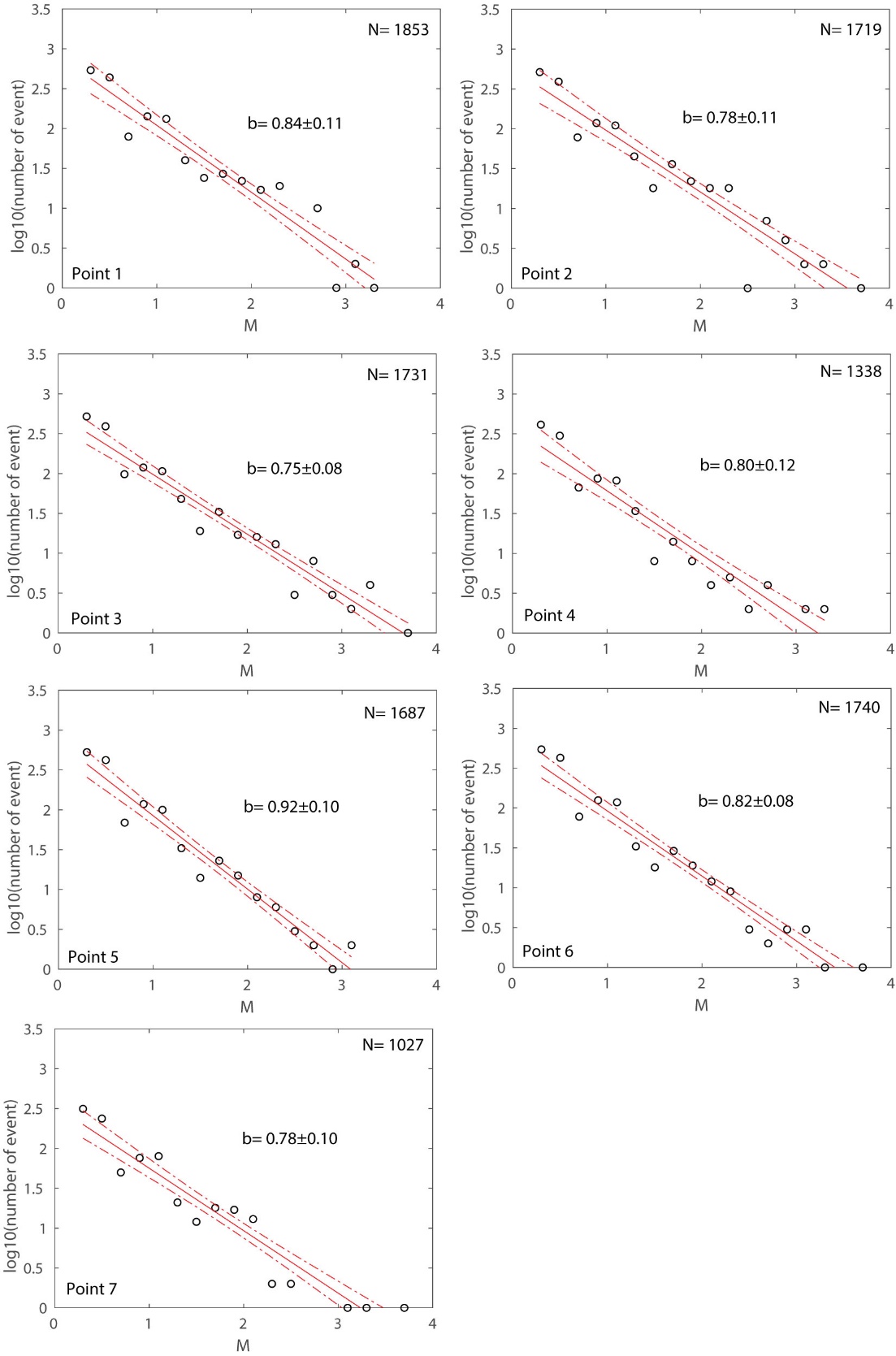


**Figure S1.** The *b*-value estimation for observation regions 1–7 for the period from 1996 to 2020. The red solid and dashed-dotted lines represent the regression line and its 90% confidence interval, respectively. The total number of earthquakes is indicated in each subfigure.

**
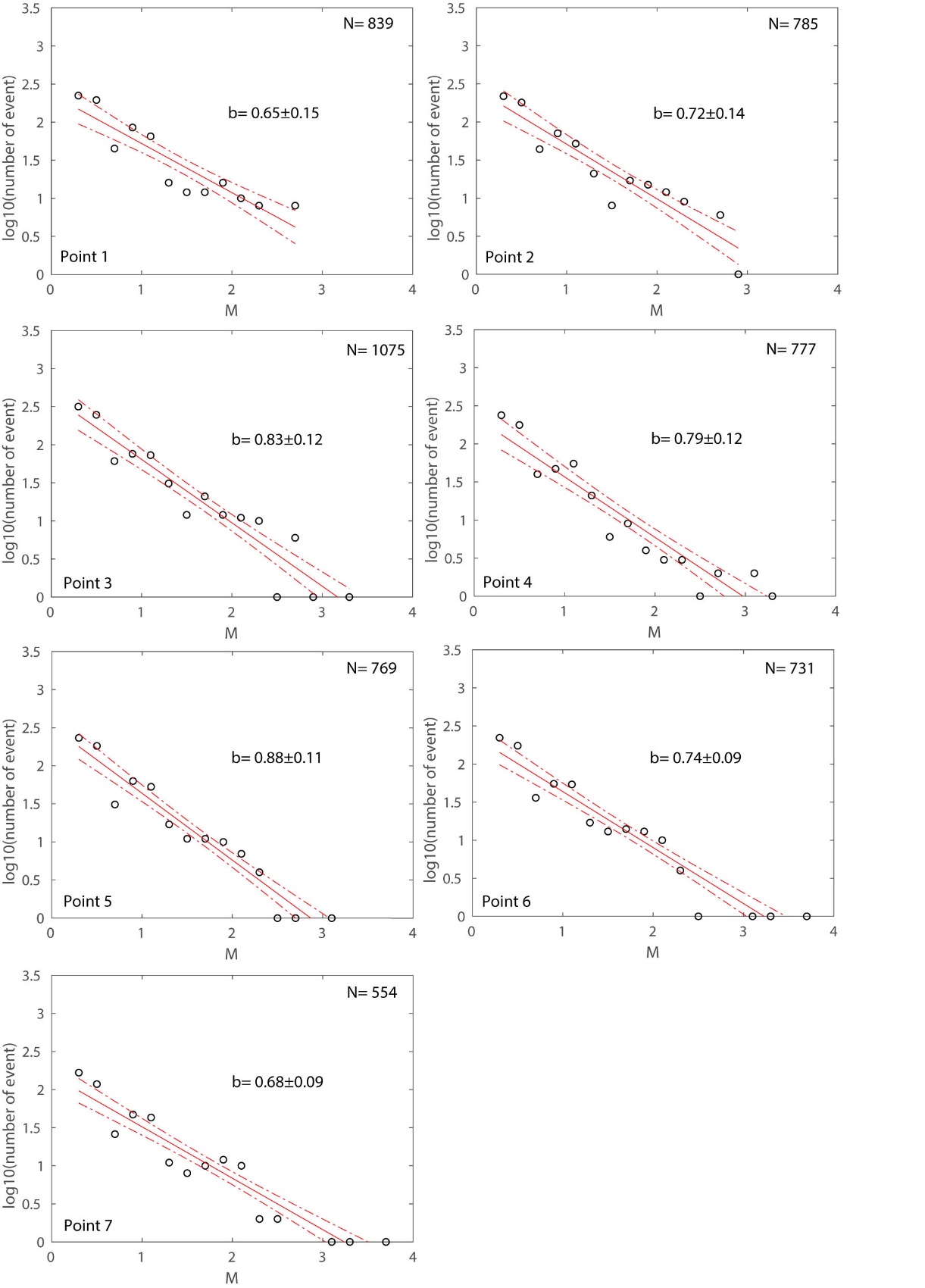
**

**Figure S2.** The *b*-value estimation for the period before the Kumamoto earthquake in regions 1–7. The red solid and dashed-dotted lines represent the regression line and its 90% confidence interval, respectively. The total amount of earthquakes is displayed in each subfigure.

**
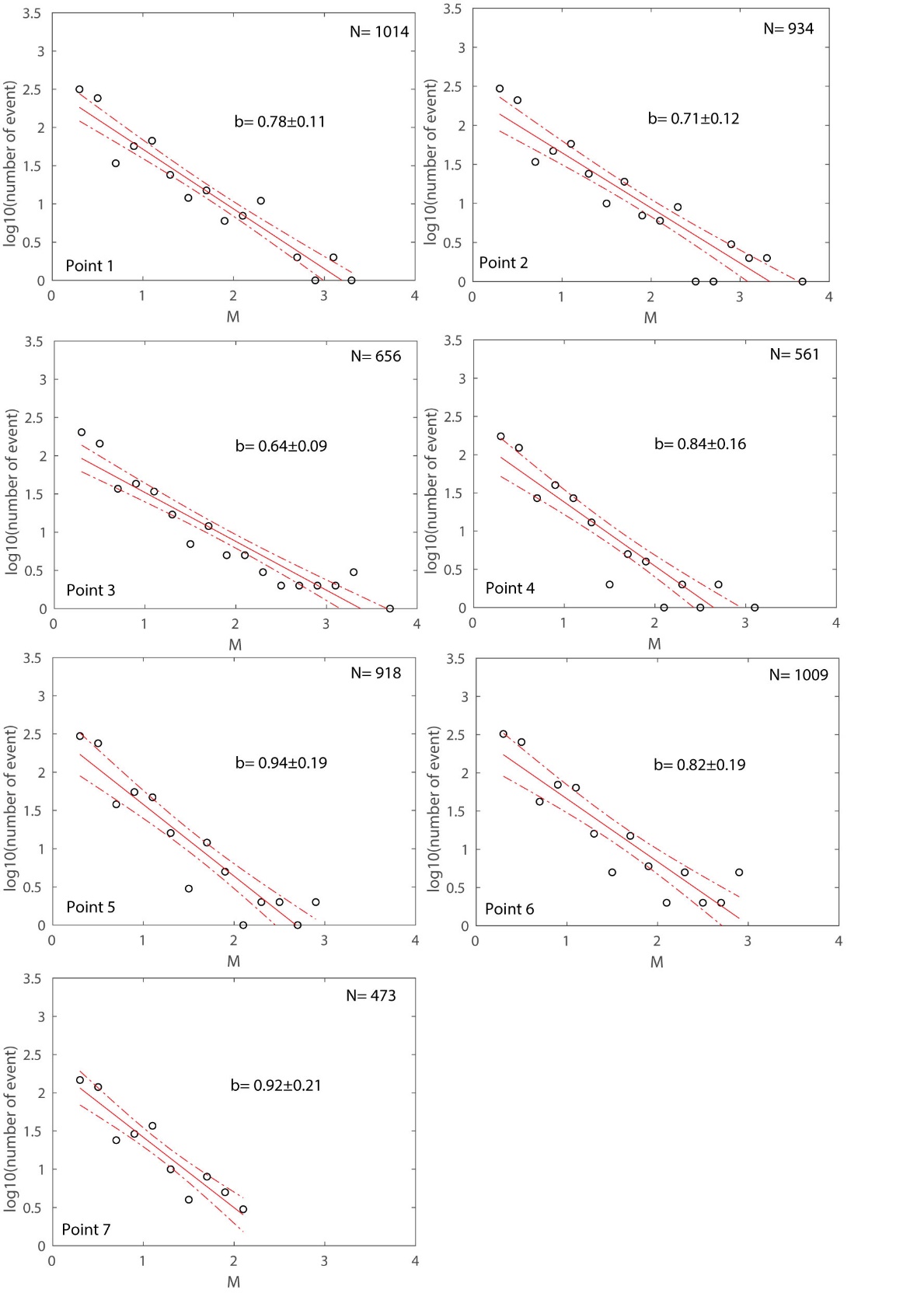
**

**Figure S3.** The b-value estimation for the period after the Kumamoto earthquake for points 1–7. The red solid and dashed-dotted lines are the regression line and its 90% confidence interval, respectively. The total amount of earthquakes is displayed in each subfigure.

**
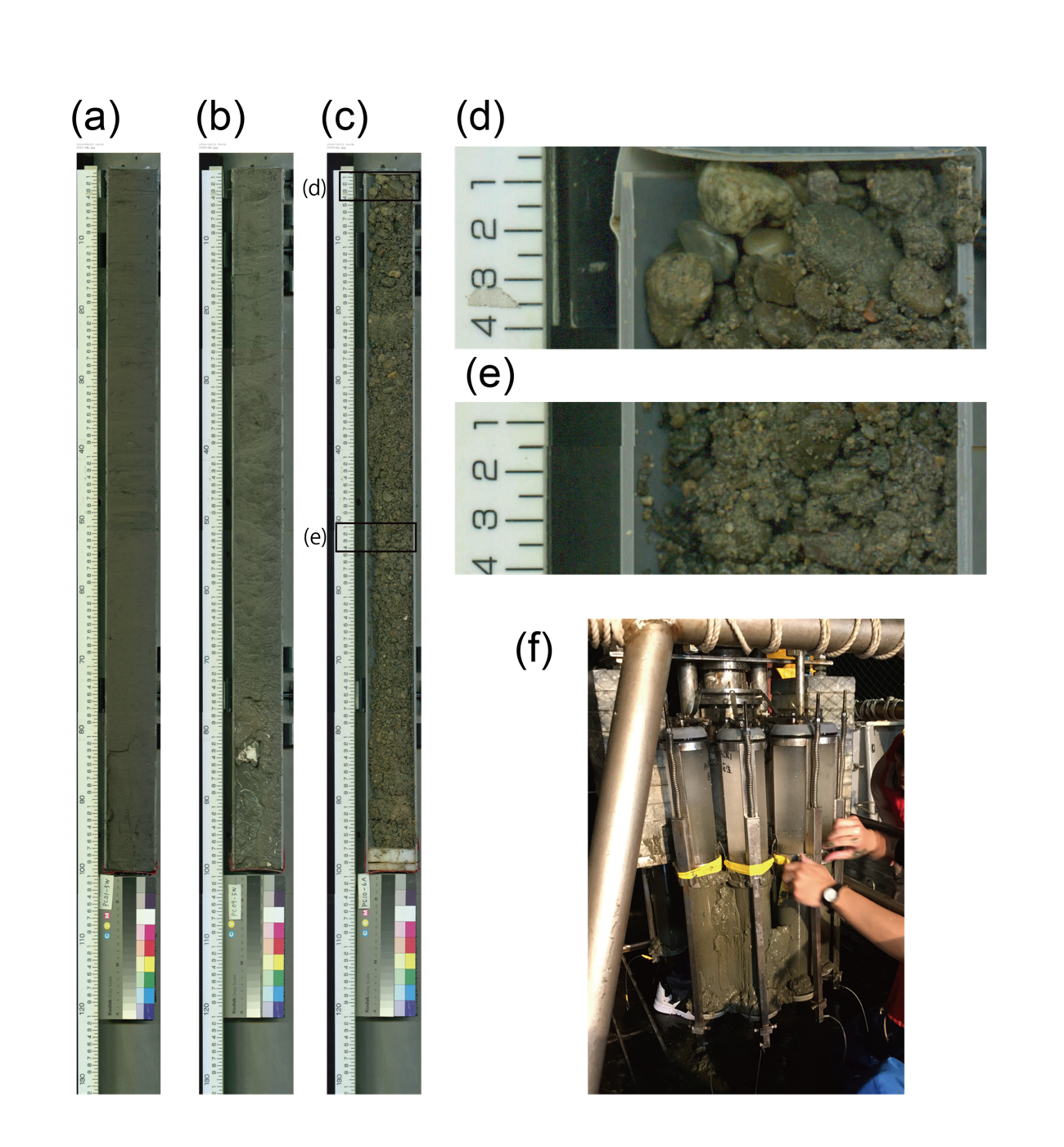
**

**Figure S4.** Photo of examples of piston cores of (a) mud, (b) sand, and (c) conglomerate, which are from PC1-5m, PC9-5m, and PC10-6m, respectively. (d) Enlarged photo from (c) shows granitoid gravels. (e) Enlarged photo from (c) displays typical sandy and muddy gravels. (f) Multiple coring on board after sampling reveals soft mud and water just above the sediment, referred to as “bottom water.”


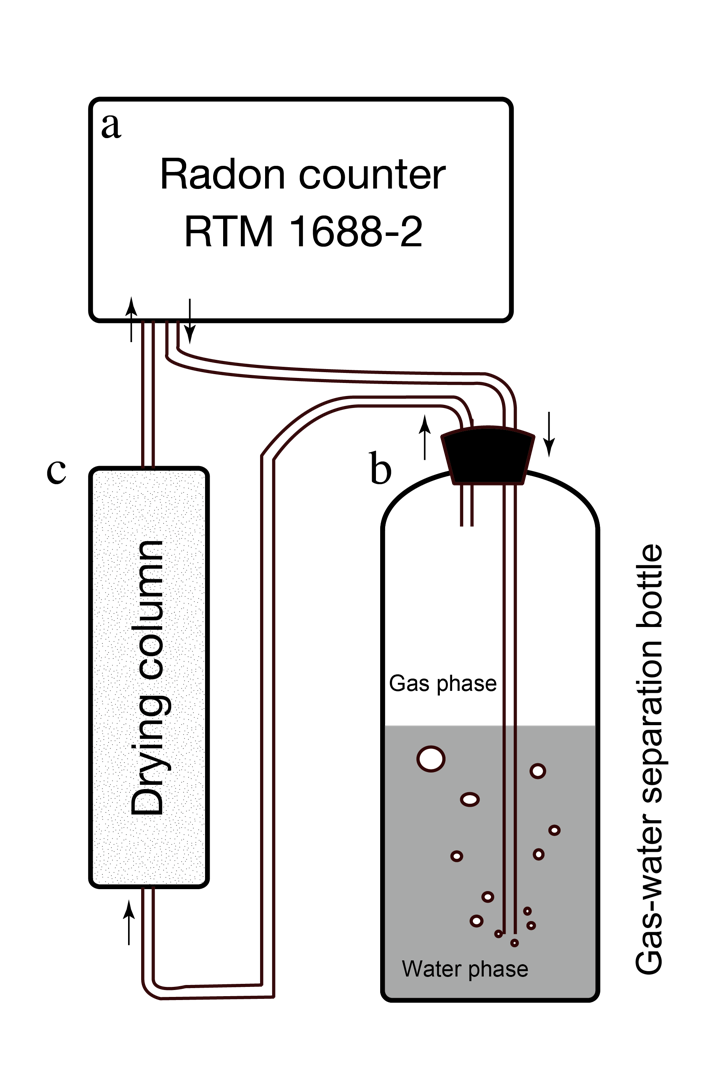


**Figure S5.** Measurement system of ^222^Rn in water. (a) Radon monitor RTM 1688, SARAD GmbH, Germany. The air in the tubes is circulated by the pump in the radon monitor. (b) Gas-water separation bottle. (c) Dehumidifier using the DRIERITE®.
